# Test–retest reliability and convergent validity of the test of nonverbal intelligence-fourth edition in patients with schizophrenia

**DOI:** 10.1186/s12888-021-03041-4

**Published:** 2021-01-13

**Authors:** Kuan-Wei Chen, Ya-Chen Lee, Tzu-Ying Yu, Li-Jung Cheng, Chien-Yu Chao, Ching-Lin Hsieh

**Affiliations:** 1 Department of Occupational Therapy, Kaohsiung Municipal Kai-Syuan Psychiatric Hospital, Kaohsiung, Taiwan; 2 Department of Occupational Therapy, College of Medical and Health Science, Asia University, Taichung, Taiwan; 3 Department of Occupational Therapy, College of Medicine, I-Shou University, Kaohsiung, Taiwan; 4 School of Occupational Therapy, College of Medicine, National Taiwan University, 4F, No.17, Xuzhou Rd., Zhongzheng Dist, Taipei City, 100 Taiwan; 5 Department of Physical Medicine and Rehabilitation, National Taiwan University Hospital, Taipei, Taiwan

**Keywords:** Cognition, Fluid intelligence, Intelligence, Psychometrics, Schizophrenia

## Abstract

**Background:**

Fluid intelligence deficits affect executive functioning and social behaviors in patients with schizophrenia. To help clinicians manage fluid intelligence deficits, a psychometrically sound measure is needed. The purposes of this study were to examine the test–retest reliability and convergent validity of the Test of Nonverbal Intelligence-Fourth Edition (TONI-4) assessing fluid intelligence in patients with schizophrenia.

**Methods:**

A total of 103 patients with stable condition were assessed with the TONI-4 twice with a 4-week interval to examine the test–retest reliability. We further used the Montreal Cognitive Assessment (MoCA) and the Tablet-Based Symbol Digit Modalities Test (T-SDMT) to examine the convergent validity of the TONI-4.

**Results:**

The intra-class correlation coefficient was 0.73 for the TONI-4. The percentages of standard error of measurement and minimal detectable change for the TONI-4 were 5.1 and 14.2%, respectively. The practice effect of the TONI-4 was small (Cohen’s d = − 0.03). Convergent validity showed small to moderate significant correlations between the TONI-4 and the MoCA as well as the T-SDMT (*r* = 0.35, *p* = .011 with the T-SDMT and *r* = 0.61, *p* < .001 with the MoCA). The results demonstrated that the TONI-4 had good test–retest reliability, limited random measurement error, and a trivial practice effect. The convergent validity of the TONI-4 was good.

**Conclusions:**

These findings indicate that the TONI-4 has potential to be a reliable and valid assessment of fluid intelligence in patients with schizophrenia.

## Introduction

Fluid intelligence can be defined as the ability to think logically and solve problems in novel situations [1, 2]. Conceptually, fluid intelligence has been linked to executive functioning and complex social behavior [3, 4]. Fluid intelligence is a critical cognitive ability affecting a wide variety of daily activities [5, 6]. Fluid intelligence deficits are common in patients with schizophrenia, and these deficits are often associated with cognitive impairment in this group [7–12]. The deficits of fluid intelligence in patients with schizophrenia are associated with difficulties in daily independent functioning [10, 11, 13]. Moreover, low fluid intelligence, which is included in the premorbid intelligence exhibited by patients with schizophrenia, precedes the first psychotic episode and appears to be related to the risk for schizophrenia [4, 8, 10, 14–17]. To help clinicians manage patients’ fluid intelligence, clinicians and researchers have to administer reliable and valid assessments of such deficits.

Three assessments are commonly used to assess fluid intelligence [4, 18–20]. They are the Comprehensive Test of Nonverbal Intelligence–Second Edition (CTONI-2) [21, 22], the Raven Advanced Progressive Matrices Test (RAPM) [23, 24], and the Test of Nonverbal Intelligence–Fourth Edition (TONI-4) [25]. However, some items of the CTONI-2 have cultural bias. For example, one item includes pictures related to American football or faces of Caucasians. In contrast, both the RAPM and the TONI-4 are administered with geometric patterns, which are not culturally dependent. Comparing the administration time, the CTONI-2 and the RAMP usually take an average of 40–60 min to finish, while the TONI-4 can be finished within 15 min. For time-pressed clinicians, the TONI-4 has great potential for assessing fluid intelligence in patients with schizophrenia.

Some supportive evidence on the psychometric properties has been found for the TONI-4 (e.g., sufficient test–retest reliability and construct validity) in healthy groups [25, 26]. However, the TONI-4 has not yet been validated in patients with schizophrenia. Because psychometric properties are generally sample dependent [27, 28], psychometric studies are needed to confirm whether the TONI-4 is reliable and valid in patients with schizophrenia. Particularly, sufficient psychometric properties (e.g., test–retest reliability, practice effect, random measurement error, and validity) are required for a measure to ensure its clinical utility for repeated assessments in patients with schizophrenia.

The current study aimed to examine the test–retest reliability, practice effect, random measurement error, and convergent validity of the TONI-4 in patients with schizophrenia. The results of the study should help clinicians and researchers determine the utility of the TONI-4 when applied to patients with schizophrenia.

## Methods

### Participants

We recruited participants via convenience sampling from a psychiatric hospital in southern Taiwan between June 2017 and April 2018. Patients were included in this study if they met the following criteria: (1) diagnosis of schizophrenia according to the Diagnostic and Statistical Manual of Mental Disorders, 5th edition [29]. DSM-5 criteria for schizophrenia was assessed and validated by board-certified psychiatrists and supported by clinical observations and interviews during hospitalization, past medical records, and information provided by main caregivers, (2) age ≥ 20 years, and (3) stable use and dosage of antipsychotic medication for at least 1 month prior to recruitment. The exclusion criteria were (1) diagnosis of other neurological or psychiatric diseases affecting cognition (e.g., stroke or depression), (2) another severe medical condition or psychiatric disorder that required treatment during the study, or (3) unstable severity of symptoms [specifically, a change in score of more than 2 on the Clinical Global Impressions Scale–Severity (CGI-S)] [30].

This study was approved by the Institutional Review Board of the local hospital. All participants signed consent forms before participating in this study.

### Procedure

This study was comprised of three assessments with 2-week intervals between adjacent assessments (i.e., early, middle and late assessments). At the early and late assessments, the participants completed alternate forms of the TONI-4 (i.e., Form A at the early assessment and Form B at the late assessment) at a four-week interval. At the middle assessment, we administered the Tablet-Based Symbol Digit Modalities Test (T-SDMT) [31] and the Montreal Cognitive Assessment (MoCA) [32]. All assessments were administered by a trained occupational therapist, using standardized protocols, forms, and manuals. In addition, the CGI-S was administered in each session to confirm that the participants’ symptoms did not change during the study period. We collected the patients’ demographic characteristics from chart review.

### Measures

#### TONI-4

The TONI-4 is designed to assess fluid intelligence in individuals aged 6 years to 89 years and 11 months. The TONI-4 has alternate forms (i.e., Form A and Form B) to reduce the practice effect [25]. Items for Form A can be found on one side of the picture book, and items for Form B are found on the reverse side. The two forms are not interchangeable. Each item is composed of a sequence of abstract figures with a figure missing from the sequence. Each sequence includes one or more attributes, such as shape, position, direction, rotation, contiguity, shading, size, and movement. Items ascend in level of difficulty as more attributes are added. When three of the five consecutive items are incorrectly answered, the test is terminated. The items are scored dichotomously: Correct answers earn one point and incorrect answers earn zero points. The rater writes the score on the answer sheet. The TONI-4 is norm referenced and yields an index, which is a standardized score (quotient) with a mean of 100 and a standard deviation of 15. Higher index scores indicate better fluid intelligence [25].

#### T-SDMT

The T-SDMT was developed from the SDMT to assess processing speed [31]. This test includes 9 different symbols, each associated with a number (1–9), presented to the examinee on a tablet computer screen (i.e., an iPad). All trials are conducted with the tablet in landscape orientation, held in place by a case that is adjusted to a 30-degree tilting angle. To respond to each item, the participant is required first to look at the symbol in the center of the screen, then to search for the corresponding number in the table at the top of the screen, and finally to choose the corresponding number on a 3-by-3 grid at the bottom of the screen. The tablet computer automatically records the number of correct answers during the test. A higher number of correct answers indicates better performance of processing speed. The T-SDMT has acceptable psychometric properties in patients with schizophrenia [31].

#### MoCA

The MoCA briefly measures overall cognitive functioning, including orientation, memory, visuospatial skills, executive functioning, language, and attention [32]. The total scores range between 0 and 30, and higher scores indicate better cognitive functioning. The total score (including the addition of one point for examinees with 12 or fewer years of education) is used for analysis. The MoCA has demonstrated high sensitivity as a cognitive screening test for severe mental illness [33].

#### CGI-S

The CGI-S assesses symptom severity on a 7-point scale (1–7) [30]. One point on the CGI-S represents that a patient is not ill, and 7 points represents most severely ill. We used the CGI-S to examine whether the symptom severity of the participants was stable during the study period.

### Statistical analyses

#### Test–retest reliability

Test–retest reliability was estimated using the intra-class correlation coefficient (ICC) between the early and late assessments, on the basis of a two-way random-effects model with absolute agreement [34]. The following criteria were used to interpret ICC values: an ICC value ≥0.80 indicated excellent test–retest reliability; 0.60–0.79, good; 0.40–0.59, moderate; and < 0.40, poor [35].

The standard error of measurement (SEM) is an index of random measurement error that can be used to present the precision of individual scores [36]. The SEM% was calculated by dividing the SEM by the mean of the early assessment score and then multiplying the result by 100% (SEM%). An SEM% of less than 10% is considered to indicate limited random measurement error for a measure [37].

We also calculated the minimal detectable change (MDC) and MDC percentage (MDC%) to examine the change between adjacent assessments that could be considered as a real change (beyond the score change caused by random measurement error) at the 95% confidence level. The MDC% was calculated by dividing the MDC by the mean of the early assessment score and then multiplying the result by 100% [38].

In addition, the agreement between test–retest measurements was analyzed by Bland–Altman plots with 95% limits of agreement (LOA) [39]. In these plots, the differences (d) between each pair of assessments were presented against the average value for each pair of assessments. To examine whether heteroscedasticity existed, Pearson’s correlation coefficient (*r*) was used to calculate the correlation between the absolute value of the difference of two assessments and the mean score of two assessments [40]. When Pearson’s *r* was ≥0.3 or ≤ − 0.3, it meant that the absolute value of the difference was related to the mean score of two assessments, and that there was heteroscedasticity [41]. In other words, the higher the assessment score, the greater (*r* ≥ 0.3) or smaller (*r* ≤ − 0.3) the difference between the two assessments.

Effect size (Cohen’s *d*) was used to estimate the magnitudes of practice effects due to repeated assessments of the TONI-4. An effect size ≥0.80 was considered as a large practice effect; 0.50–0.79, medium; 0.20–0.49, small; and < 0.20, trivial [42].

To further examine whether the findings were consistent across participants’ genders and ages, sub-group analysis was performed. We stratified the participants by gender and three age bands (i.e., 20–39, 40–49, and 50–70) individually.

#### Convergent validity

Convergent validity was examined by correlating the scores of the TONI-4 at the early assessment with those of the MoCA and the T-SDMT using Pearson’s *r*. We hypothesized that we would find moderate correlations between the scores of the TONI-4 and the MoCA (i.e., fluid intelligence and cognition) [43], and that small correlations would be found between the scores of the TONI-4 and the T-SDMT (i.e., fluid intelligence and processing speed) [3, 18].

## Results

We recruited 106 patients with schizophrenia who were eligible for the study. Of these, 103 participants completed all assessments. About half of the participants were male (50.5%), and the mean age was 46.7 years. The demographic and clinical characteristics of the participants are shown in Table 1. The early and late assessments scores of the TONI-4, on average, were very similar (92.4 and 91.9), indicating that the participants had slight impairment of fluid intelligence. In addition, the mean score of the MoCA was 23.3, indicating that our participants, on average, had mild cognitive impairment. The mean score of the T-SDMT was 32.1, indicating that the processing speed of most participants was impaired.

**Table 1:** Demographic characteristics of the participants (*n* = 103)

Characteristic	Value
Gender, n (%)	
Male	52 (50.5%)
Female	51 (49.5%)
Age range, years	
Age, mean (SD)	46.7 (10.2)
20–39	28 (27.2%)
40–49	30 (29.1%)
50–70	45 (43.7%)
Age of onset, years, mean (SD)	26.5 (8.3)
Duration of illness, years, mean (SD)	20.2 (9.3)
Education level, n (%)	
Illiterate	0
Elementary	7 (6.8%)
Middle school	38 (36.9%)
High school or vocational school	41 (39.8%)
University or graduate school	17 (16.5%)
Marital status, n (%)	
Married	28 (27.2%)
single	71 (68.9%)
Other	4 (3.9%)
CGI-S (SD)	3 (0.8)
MoCA (SD)^a^	23.3 (4.6)
T-SDMT (SD)^a^	32.1 (9.1)

^a^There were some missing data in the variables of the MoCA and T-SDMT

Note: *CGI-S* Clinical Global Impressions Scale-Severity, *MoCA* Montreal Cognitive Assessment, *T-SDMT* Tablet-Based Symbol Digit Modalities Test

### Test–retest reliability

Table 2 shows the results of the test–retest reliability analyses. The ICC of the TONI-4 was 0.73 (95% confidence interval: 0.62 to 0.81).

**Table 2:** The mean, SD, ICC, Cohen’s d, SEM and MDC of the TONI-4 (*n* = 103)

TONI-4	Early assessment Mean (SD)	Late assessment Mean (SD)	Cohen’s d	ICC (95% CI)	SEM (SEM%)	MDC (MDC%)
Sum score	92.4 (9.1)	91.9 (9.9)	−0.03	0.73 (0.62–0.81)	4.7 (5.1)	13.1 (14.2)
Age band						
20–39	92.5 (8.7)	93.8 (10.4)	0.13	0.70 (0.45–0.85)	4.7 (5.1)	13.2 (14.2)
40–49	92.2 (9.6)	89.3 (9.7)	−0.29	0.64 (0.37–0.81)	5.8 (6.3)	16.0 (17.3)
50–70	92.4 (9.2)	92.5 (9.6)	0.00	0.82 (0.69–0.90)	3.9 (4.2)	10.9 (11.7)
Gender						
Male	92.3 (9.3)	93.4 (9.7)	0.12	0.70 (0.53–0.81)	5.1 (5.5)	14.1 (15.3)
Female	92.4 (8.9)	90.4 (10.0)	−0.21	0.76 (0.61–0.86)	4.4 (4.7)	12.1 (13.1)

Note: *SD* Standard deviation, *ICC* Intra-class correlation coefficient, *SEM* Standard error of measurement, *SEM%* Percentage of standard error of measurement, *MDC* Minimal detectable change, *MDC%* Percentage of minimal detectable change

The SEM (SEM%) and MDC (MDC%) of the TONI-4 scale were 4.7 (5.1%) and 13.1 (14.2%) points, respectively. The results were smaller than our preset criterion.

**Fig. 1:**
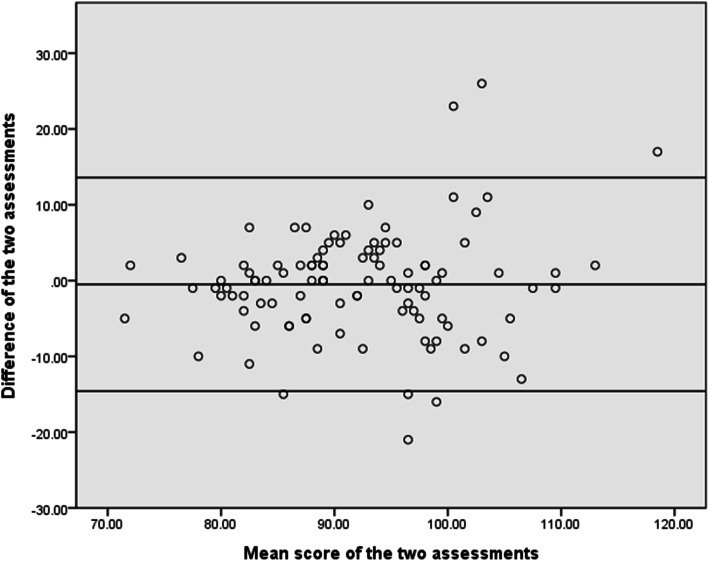
Bland–Altman plot showing the difference scores against the mean scores of pair of scores on the TONI-4. Note. The solid line shows the mean of the differences (− 0.5). The two dashed lines represent 95% limits of agreement (d ± 1.96 × SD = −14.6–13.6)

In Fig. 1, the LOAs ranged from − 14.6 to 13.6 points. Pearson’s *r* between the absolute value of the difference of the early and late assessments and the mean score of the early and late assessments was 0.31.

Analysis of the practice effect revealed that the effect size of score change in the TONI-4 was small (Cohen’s *d* = − 0.03) between the early and late assessments.

To further examine whether the aforementioned findings were consistent across participants’ genders (male and females) and ages (20–39, 40–49, and 50–70), sub-group analysis was performed. The results showed that the ICCs (0.64–0.82), SEM%s (4.2–6.3%), MDC%s (11.7–17.3%), and Cohen’s ds (− 0.29–0.13) of the TONI-4 were similar across all sub-groups.

### Convergent validity

The index scores of the TONI-4 were moderately correlated with the scores of the MoCA (*r* = 0.61, *p* < .001, *n* = 96), whereas a small correlation was found between the scores of the TONI-4 and the T-SDMT (*r* = 0.35, *p* = .011, *n* = 51). In addition, there were no significant differences in the scores of the TONI-4 between the participants who had and those who had not been assessed with the MOCA (t = − 4.82, *p* = 0.631) and the T-SDMT (t = − 4.29, *p* = 0.669).

## Discussion

### Test–retest reliability

A measure with sufficient test–retest reliability ensures that users can obtain reproducible scores. Good test–retest reliability was found for the repeated assessments of the TONI-4. Moreover, the test–retest reliabilities were similar across the gender and age sub-groups. Accordingly, the TONI-4 has generally good test–retest reliability, which may not be affected by examinees’ gender and age, and it can be used in repeated assessments. In comparison with previous studies, the test–retest reliability of our study was slightly lower than those found for healthy controls (*r* = 0.82–0.93) [25] and was consistent with those of other cognitive assessments examining patients with schizophrenia [44, 45]. There are three possible reasons for the slightly lower ICC of the TONI-4. First, the test–retest reliability was estimated by Pearson correlation coefficients in the previous studies, which tends to overestimate reliability [34]. Second, alternate forms (i.e., Forms A and B) were used in this study, which may have resulted in more variation compared to using the same form as previous studies [25]. Third, the heterogeneity of our sample appeared limited. In particular, the variances of the TONI-4 in this study (SDs = 9.1 and 9.9) were smaller than those of a previous study (SDs = 13–15) [25], which may have underestimated the ICC values in this study [46]. In summary, our findings indicate that the TONI-4 appears to be reliable for repeatedly assessing fluid intelligence in patients with schizophrenia.

We found that the SEM% was far below our preset criterion. Furthermore, the SEM%s were generally consistent across the gender and age sub-groups. These findings suggest that the TONI-4 has limited random measurement error. Our findings are consistent with those in previous studies examining healthy groups, where the SEM% were 4.0–5.5% [25]. These findings support that the random measurement error is similar in patients with schizophrenia and in healthy adults. Therefore, the scores of the TONI-4 tend to be stable in patients with schizophrenia.

In addition, MDC can be viewed as the threshold for a statistically significant change for individual patients in clinical and research settings [47]. Conceptually, a change exceeding the MDC of the first assessment can be interpreted as a real improvement with the corresponding certainty (e.g., 95%). Thus, a fixed MDC value can be used to interpret the change scores for patients with different levels of fluid intelligence. However, we found that the association between the absolute value of the difference of the early and late assessments and the mean score of the early and late assessments (Pearson’s *r* = 0.31) was above 0.30, implying the existence of heteroscedasticity [41]. That is, the absolute difference and the mean of the early and late assessments increased simultaneously. Accordingly, a fixed value of MDC is not appropriate for different levels of fluid intelligence.

In such assessments with heteroscedasticity, the MDC% is more suitable than the MDC for interpreting a true change for a patient [48]. That is, as seen in this study, the MDC value can be adjusted based on the MDC% and the patient’s early assessment score. Specifically, the MDC% (14.2%) of the TONI-4 can be multiplied by the patient’s early assessment score to achieve an adjusted MDC value. For example, a patient with a score of 92 points at the early assessment requires an improvement of more than 13.1 points (92 × 0.142) to indicate a true change. These adjustments can help clinicians and researchers interpret the score changes on the TONI-4 of an individual patient after intervention and then develop further treatment plans accordingly.

We found that the scores between the early and late assessments had almost no change. In addition, those values were similar across the sub-groups of examinees’ gender and age. These findings indicate that the scores of the TONI-4 do not systematically increase given that the early assessment (or practice) has already been completed. Our findings are consistent with those in a previous study, where the change scores within one-to-two-week intervals were small (effect size = 0.00–0.07) [25]. The trivial practice effect may have been due to the use of alternate forms (i.e., Forms A and B) [49, 50]. However, using alternate forms may lead to underestimation of the practice effect as compared to using a single form. In this study, all participants were administered the forms in a fixed order (i.e., Form A first and Form B second). The fixed order design was used because previous findings had indicated that test–retest reliability is not affected by the order effect [26]. Thus, clinicians could use alternate forms of the TONI-4 in their routine repeated assessments to effectively minimize practice effects.

### Convergent validity

We found that the scores of the TONI-4 were moderately correlated with those of the MoCA and significantly correlated with those of the T-SDMT, supporting our hypotheses. Thus, good convergent validity was demonstrated for the TONI-4. Our results support the validity of the TONI-4 for assessing fluid intelligence in patients with schizophrenia.

This study had two merits. First, the sample size (103 participants) was relatively large. A large sample size tends to provide robust estimates, which improves the generalizability of our findings [51]. Second, we used alternate forms of the TONI-4. Due to this study design, the practice effects of the TONI-4 were well controlled, so its utility in repeated assessments was confirmed.

### Implications

The fluid intelligence encompasses the ability to think logically and solve problems in novel situations, which is a critical cognitive ability affecting clients’ performance on a wide variety of daily activities. Knowledge and evidence of the test–retest reliability and convergent validity of the TONI-4 help clinicians select a measure for assessing fluid intelligence in patients with schizophrenia.

- The TONI-4 appears reliable for repeatedly assessing the fluid intelligence in patients with schizophrenia.
- Due to heteroscedasticity of the TONI-4, an adjusted MDC, the patient’s early assessment score multiplied by the MDC% (14.2%), is suggested for use in determining whether the change in score of a patient is outside the range of random measure error.
- The good convergent validity of the TONI-4 provides a preliminary basis to support its utility for assessing fluid intelligence in patients with schizophrenia.

### Study limitations

Two limitations of this study should be noted. First, the study sample was a convenience sample recruited from a psychiatric center in southern Taiwan. In addition, our participants, on average, had slightly impaired fluid intelligence (the mean score of the TONI-4 was 92.4 points at the early assessment). The above sampling limitations might have affected the generalizability of our findings. Second, we used alternate forms to examine the test–retest reliability of the TONI-4. Thus, our results on test–retest reliability might not be generalizable to single-form assessment of the TONI-4. Using alternate forms may lead to underestimation of the test–retest reliability as compared to using a single form.

## Conclusions

We found good test–retest reliability and good convergent validity of the TONI-4 in patients with schizophrenia. These findings provide preliminary evidence supporting the utility of the TONI-4 in patients with schizophrenia.

## Data Availability

The (anonymized) datasets analyzed during the current study are available from the first author on reasonable request.

## Abbreviations

CTONI-2: Comprehensive Test of Nonverbal Intelligence–Second Edition
RAPM: Raven Advanced Progressive Matrices Test
TONI-4: Test of Nonverbal Intelligence–Fourth Edition
DSM-5: Diagnostic and Statistical Manual of Mental Disorders, 5th edition
CGI-S: Clinical Global Impressions Scale–Severity
T-SDMT: Tablet-Based Symbol Digit Modalities Test
MoCA: Montreal Cognitive Assessment
ICC: Intra-class correlation coefficient
SEM: Standard error of measurement
MDC: Minimal detectable change
LOA: Limits of agreement
SD: Standard deviation
